# Gray-matter structure in long-term abstinent methamphetamine users

**DOI:** 10.1186/s12888-020-02567-3

**Published:** 2020-04-10

**Authors:** Lili Nie, Zeyong Zhao, Xiantao Wen, Wei Luo, Tao Ju, Anlian Ren, Binbin Wu, Jing Li

**Affiliations:** 1 Mental Health Center, West China Hospital of Sichuan University, Chengdu, 610041 China; 2 Detoxification and Narcotics Control Department of Sichuan Province, Chengdu, 610041 China; 3 Sichuan provincial Compulsory Drug Addiction Treatment Agency for Males, Ziyang, 641400 China; 4 Sichuan provincial Compulsory Drug Addiction Treatment Agency for Females, Deyang, 618007 China; 5 Hospital of Sichuan provincial Compulsory Drug Addiction Treatment Agency for Females, Deyang, 618007 China

**Keywords:** Methamphetamine, Abstinence, Magnet resonance imaging, Gray-matter, Volume, Thickness

## Abstract

**Background:**

Previous studies of brain structure in methamphetamine users have yielded inconsistent findings, possibly reflecting small sample size and inconsistencies in duration of methamphetamine abstinence as well as sampling and analyses methods. Here we report on a relatively large sample of abstinent methamphetamine users at various stages of long-term abstinence.

**Methods:**

Chronic methamphetamine users (*n* = 99), abstinent from the drug ranging from 12 to 621 days, and healthy controls (*n* = 86) received T1-weighted structural magnetic resonance imaging brain scans. Subcortical and cortical gray-matter volumes and cortical thickness were measured and the effects of group, duration of abstinence, duration of methamphetamine use and onset age of methamphetamine use were investigated using the Freesurfer software package.

**Results:**

Methamphetamine users did not differ from controls in gray-matter volumes, except for a cluster in the right lateral occipital cortex where gray-matter volume was smaller, and for regions mainly in the bilateral superior frontal gyrui where thickness was greater. Duration of abstinence correlated positively with gray-matter volumes in whole brain, bilateral accumbens nuclei and insulae clusters, and right hippocampus; and with thickness in a right insula cluster. Duration of methamphetamine use correlated negatively with gray-matter volume and cortical thickness of a cluster in the right lingual and pericalcarine cortex.

**Conclusions:**

Chronic methamphetamine use induces hard-to-recover cortical thickening in bilateral superior frontal gyri and recoverable volumetric reduction in right hippocampus, bilateral accumbens nuclei and bilateral cortical regions around insulae. These alternations might contribute to methamphetamine-induced neurocognitive disfunctions and reflect a regional specific response of the brain to methamphetamine.

## Background

Amphetamine-type stimulants contribute substantially to the global burden of disease from drugs of abuse, ranking second only to opioids in this regard [1]. Among them, methamphetamine is the most widely used [1], and acts in part through promoting release of dopamine and serotonin [2]. Administration of methamphetamine produces long-term damage to dopaminergic and serotonergic neurons [2, 3], which project from their cell bodies to remote targets [4], such as the striatum, hippocampus, and prefrontal cortex [5], where chronic methamphetamine exposure and subsequently abstinence would be expected to produce structural changes. T1-weighted magnet resonance imaging (MRI) has been combined with voxel-based morphometry to measure regional gray-matter volumes to address this question, but with inconsistent results.

Studies on active chronic users directly provides evidences on the effects of chronic methamphetamine use. Available reports generally indicate that subcortical gray-matter structures are larger, and that cortical volumes are smaller in this population [6]. Larger volumes have been observed in bilateral putamen [7, 8] and left nucleus accumbens [8], but smaller volume was measured in the hippocampus [9]. Smaller cortical volumes have been measured throughout the cerebral cortex, specifically in these regions: left superior frontal gyrus [8], left precentral gyrus [8, 10], right inferior parietal cortex [7], right supramarginal gyrus [10], left superior and right inferior temporal gyri [10], right superior lateral occipital cortex [8], right anterior cingulate cortex, post cingulate cortex and paralimbic belts [9], and in the right dorsolateral cerebellum [7]. Of these findings, only greater volume in right putamen and smaller volume in left precentral gyrus were found in more than one sample.

Studies on abstinent chronic users provide evidences on the effects of abstinence. However, available reports are insufficient for a comprehensive conclusion. At 18 days of abstinence, smaller volumes were observed in dorsolateral prefrontal and orbitofrontal cortices, as well as in subregions of the superior temporal gyrus [11]. At 2 months of abstinence, smaller volumes were observed in bilateral insulae and left middle frontal gyrus [12], but larger volume in part of the cerebellum cortex was also observed [12]. When the period of abstinence prolonged to 3 months, greater volumes of the caudate and accumbens nuclei, putamen, globus pallidus and parietal lobe were observed [13]. At 4 months of abstinence, greater putamen and globus pallidus volume was observed [14]. At 6 months of abstinence, smaller volumes in right precentral, left fusiform gyri, the head of the caudate nucleus and in right cerebellum cortex were observed [15]. When the abstinence prolonged to 20 months, smaller volume in right middle frontal and inferior frontal gyri were observed [16].

Of these findings, only the greater volume of putamen and accumbens nuclei were observed in active users, and only greater volumes of the putamen and globus pallidus were replicated in two samples with similar abstinence periods of 3 [13] and 4 months [14]. Converging to observations at different length of abstinence, length of abstinence from methamphetamine was associated with greater volume of amygdalae [12]. Only two longitudinal studies that directly examined the effect of abstinence on gray-matter measurements also yielded discrepancy findings, although they observed different stage of abstinence. One of the studies found a widespread increase in gray-matter volume during the first month of abstinence from methamphetamine, involving bilateral frontal, temporal and parietal cortices, right insula and left occipital pole, with a concomitant decrease bilaterally in the cerebellum [10]. In another study, gray-matter volume increased in the cerebellum but decreased in the cingulate gyrus from 6 months abstinence on average to 2 months later [15].

Possible reasons for discrepancies in the literature are small sample size, which ranged from 17 [8] to 61 [12], participants’ use of drugs other than methamphetamine, and differences in data acquisition and analysis. In the present study of gray-matter structure in chronic methamphetamine users, we had a sample of methamphetamine users larger than in previous morphological studies of methamphetamine effects. The participants were relatively pure methamphetamine users and had supervised abstinence over a period of up to almost 2 years. We also used surface-based methods to perform the analyses, including cortical thickness [17] and volume measurements. This method uses spatial intensity gradients across tissue classes to create the structural maps and therefore the maps are not restricted to the voxel resolution of the original data. Thus, this method is capable of detecting submillimeter differences between groups. Based on the literatures reviewed above, we hypothesized that the measures of gray-matter in methamphetamine users would differ to that in the healthy controls, and would change along with the prolonging of abstinence. Specifically, it was expected that gray-matter increment would be observed in subcortical structures, such as striatum and hippocampus, and gray-matter loss would be observed in cortical regions, such as frontal, parietal, temporal and cingulum cortex. Additionally, it was expected that the alternations would show a recovery trend along with the prolonging of the abstinence.

## Methods

### Participants and procedure

Ninety-nine participants with a history of chronic methamphetamine use were recruited from two Compulsory Drug Addiction Treatment Agencies in China, where they maintained supervised abstinence. They were required to be between 18 and 50 years of age, to have self-administered methamphetamine at least ten times, to be taking no prescribed medications, to be able to read and write, and to have completed at least 6 years of formal education. The exclusionary criteria for this study were: 1) comorbid psychiatric disorders, including schizophrenia and bipolar disorder, 2) serious medical conditions, risk of suicide or violent behavior, 3) conditions that would render MRI unsafe for the participant or that would interfere with MRI data interpretation, 4) current pregnancy or lactation (females only).

Eighty-six sex and age matched healthy control participants with no history of addictive drug (other than alcohol and tobacco) use were recruited from the local community by advertisements (Table 1). Control participants fulfilled the same inclusion and exclusion criteria as the methamphetamine users except the history of addictive drug use.

**Table 1:** Characteristics of the participants

	Controls (*n* = 86)	MA users (*n* = 99)	X^2^/T	*p*
Sex, n, male/female	49/37	51/48	0.55	0.457
Ethnicity, n, Chinese Han /Others	80 /6	98 /1	Fisher’s exact *p* value	0.051
Smokers, n, no /yes	75 /11	29 /70	63.5	< 0.001
Age at time of study, yr	28.55 ± 8.56 (18–46)	26.95 ± 6.22 (19–50)	1.43	0.154
Full-time education, yr	14.58 ± 3.64	8.76 ± 2.95	11.839	< 0.001
Duration of MA use, months		56.49 ± 35.54 (3–142)		
Abstinence before MRI scan, days		240.96 ± 182.68 (12–621)		
Age at onset of MA use, yr		21.73 ± 6.65		
History of other substance use, n ^**a**^		27		
MDMA/ Ketamine / Both		2/21/4		

^**a**^ Total consumption of MA exceeded 90% of one’s total substance consumption (in number of administrations). All denied having used other substances including marijuana, cocaine, heroin, pethidine, morphine, methadone, codeine. During the abstinent period, smoking was limited to less than one cigarette per day, and alcohol was forbidden. MA: methamphetamine. MDMA: methylenedioxymethamphetamine

All participants received a structured interview by a certified psychiatrist. The interview provided data regarding duration of methamphetamine use, combination use of other substances, age at first use of methamphetamine, and duration of present episode of abstinence. Having ever used methamphetamine was verified by urine toxicology which was performed immediately after the participants entered the agencies. History of methamphetamine use was based on self-report. The abstinence was ensured by the agencies where the participants resided in for accepting mandatory detoxification. These agencies are run by law enforcement. Comorbid psychiatric disorders were independently identified by two psychiatrists using the Structured Clinical Interview from the Diagnostic and Statistical Manual of Mental Disorders, Fourth Edition (SCID) [18], No alcohol was used and smoking was limited to < 1 cigarette per day during the abstinence.

### Magnetic resonance image acquisition

All participants underwent a structural MRI scan using a Siemens Trio 3.0 T tomograph (Siemens Medical Solutions, Erlangen, Germany). A high-resolution T1-weighted anatomical magnetically prepared rapid acquisition gradient echo (MPRAGE) sequence was used (176 slices, 1-mm thick, TR = 1900 ms, TE = 2.26 ms, TI = 900 ms, flip angle = 9°, FOV = 256 mm × 256 mm, imaging matrix = 256 × 256), yielding 1 mm^3^ isotropic voxel resolution.

### MRI data processing

Cortical reconstruction and volumetric segmentation were performed using the Freesurfer image analysis suite (5.3.1), which is documented and freely available for download online (http://surfer.nmr.mgh.harvard.edu/). Briefly, after the removal of non-brain tissue and Talairach transformation, the image was segmented and labeled with gray-matter, white matter and CSF. Then, intensity normalization and topology correction were performed and the surfaces were extracted. The quality of the output images of each preprocessing step was manually checked and mistakes were amended.

When these steps of preprocess were conducted, the volume of each subcortical gray-matter structure were given for each participant. And, a matrix representing the thickness, which was calculated as the closest distance from the gray/white boundary to the gray/CSF boundary at each vertex on the surface [17], was also generated for each participant.

To improve the ability to detect differences between samples, we blurred each participant’s morphometric parameter map using a 10-mm full-width at half maximum surface-based Gaussian kernel.

### Statistical analysis

Demographic data were analyzed with SPSS by t test or X^2^ test as appropriate. Variables were shown as means ±SD. Group comparisons on volume of subcortical gray-matter structures used analysis of covariance (ANCOVA) controlling for age, sex, and total intracranial volume (ICV). Effects of duration of abstinence (controlling for age, sex, ICV, onset age and duration of methamphetamine use), duration of methamphetamine use (controlling for age, sex, ICV, duration of abstinence and onset age of methamphetamine use) and onset age of methamphetamine use (controlling for age, sex, ICV, duration of abstinence and duration of methamphetamine use) on volume of subcortical gray-matter structures were investigated using partial correlations. The reason why years of education was not controlled whereas ICV was controlled was clarified in the supplementary materials. Smoking and alcohol were not used as cofactors because smoking was limited to less than one cigarette per day and alcohol was forbidden in the methamphetamine users. The criterion for statistical significance was *p* < 0.05.

Whole-brain analyses (group comparisons and correlations) on the surface-based thickness/volume of the cortex were conducted in Freesurfer using ANCOVA or regression model with the covariates and cofactors controlling for, same as the analyses on subcortical gray-matter structures. The criterion for statistical significance was *p* < 0.05, corrected for multiple comparisons by Monte Carlo Null-Z Simulation (5000 times, cluster *p* < 0.05). Values of significant cortical clusters were extracted for validating the significances and plotting the scatters.

## Results

### Characteristics of the participants (Table 1)

A total of 99 (51 M/48F, 98 Chinese Han ethnicity) chronic methamphetamine users were included in this study. Their age was 26.95 ± 6.22 years old, with 8.76 ± 2.95 years of formal education. 70 of them had ever smoked cigarette. The duration of methamphetamine use was 56.49 ± 35.54 months, duration of abstinence was 240.96 ± 182.68 days, and the onset of methamphetamine use was at their 21.73 ± 6.65 years old. Of them, 2 had ever used methylenedioxymethamphetamine (MDMA), 21 had ever used ketamine and 4 had ever used both. 86 healthy controls (49 M/37F, 80 Chinese Han ethnicity) were included in this study. Their age was 28.55 ± 8.56 years old, and they accepted 14.58 ± 3.64 years of formal education. 11 of them had ever smoked cigarette. Large proportion of methamphetamine users had ever smoked cigarette (*p* < 0.001). Users accepted shorter formal education (*p* < 0.001). Other parameters of the two groups were comparable.

### Group comparisons on volume of subcortical gray-matter structures, cortical volumes and thicknesses

**Fig. 1:**
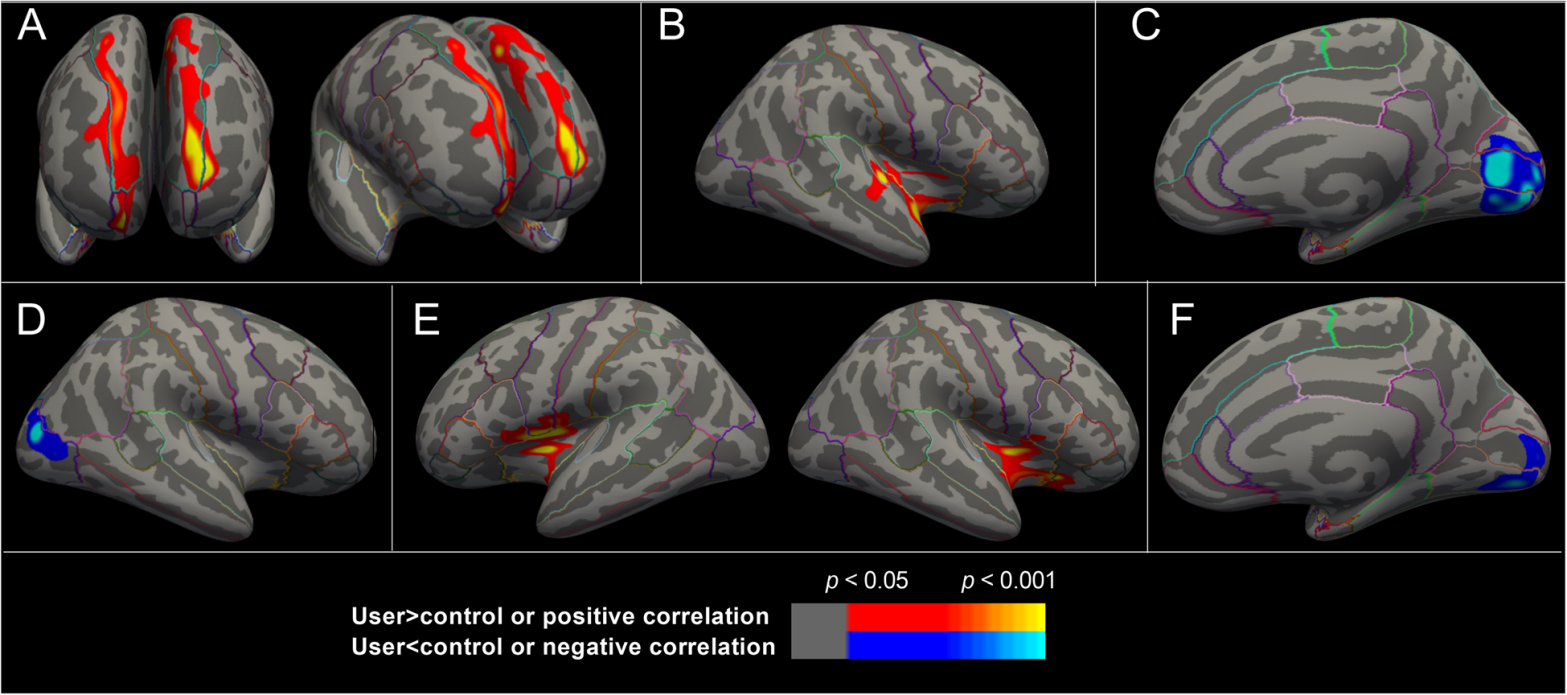
Significant cortical clusters identified in group comparisons or within-group correlation analyses. A. Greater thickness in methamphetamine (MA) users (*n* = 99) than healthy controls (*n* = 86). B. Positive correlation of duration of abstinence with cortical thickness in MA users. C. Negative correlation of duration of MA use with cortical thickness in MA users. D. Smaller volume in MA users than healthy controls. E. Positive correlation of duration of abstinence with gray-matter volume in MA users. F. Negative correlation of duration of MA use with gray-matter volume in MA users. Statistical methods were shown in the notes of Table 2. Color bar shows statistical significances. The threshold was set at vertex-wise *p* < 0.05 corrected for multiple comparisons using Monte Carlo Null-Z Simulation (5000 times, cluster *p* < 0.05)

No group difference was identified in volume of total gray-matter and subcortical gray-matter structures (*p*s > 0.05) (Supplementary Table 1). The volume of a cluster in right lateral occipital region was smaller in users than in controls (Table 2 and Fig. 1).

**Table 2:** Significant cortical regions identified in group comparisons or within-group correlation analyses

	Peak *p*_vertex_	*p*_cluster_	Size (mm^2^)	Talairach coordinates			Annotations
				X	Y	Z	
**Group comparisons**^**a**^ ***Thickness***							
User>Control	0.00016	0.010	1038.38	−20	59.3	1.1	L-rostralmiddlefrontal
	0.00097	0.004	1181.39	−9.7	18.7	41.5	L-superiorfrontal
	0.00061	0.0001	1659.83	7.1	56.9	−14.8	R-frontalpole, R-superiorfrontal
***Volume***							
User<Control	0.00016	0.043	864.62	33.4	−75.4	10.2	R-lateraloccipital
**Correlations**							
Thickness-ABS ^b^	0.00015(+)	0.004	1230.94	45.2	−2.7	−16.4	R-superiortemporal, R-insula
Volume-ABS ^b^	0.00007(+)	0.004	1183.08	−36.5	−0.4	15.2	L-insula
	0.00074(+)	0.009	1091.21	38.1	−0.1	1.8	R-insula
Thickness- MA use ^c^	0.00019(−)	0.0001	2796.16	5.7	− 87.8	−0.2	R-lingual, R-pericalcarine
Volume-MA use ^c^	0.00126(−)	0.009	1101.37	12.4	−79.7	−2.8	R-lingual, R-pericalcarine

Whole-brain vertex-wise analyses. Vertex-wise *p* < 0.05, corrected for multiple comparisons using Monte Carlo Null-Z Simulation (5000 times, cluster *p* < 0.05). (+) or (−) showed positive or negative correlation respectively

^a^ ANCOVA with sex, age and total intracranial volume (ICV) controlled

^**b**^ Partial correlation with sex, age, age at onset of methamphetamine (MA) use, duration of MA use (MA use) and ICV controlled

^**c**^ Partial correlation with sex, age, age at onset of MA use, duration of abstinence from MA (ABS) and ICV controlled

The thickness of three clusters mainly in the bilateral superior frontal gyri was greater in users than that in controls (Table 2 and Fig. 1).

### Effects of duration of abstinence

**Fig. 2:**
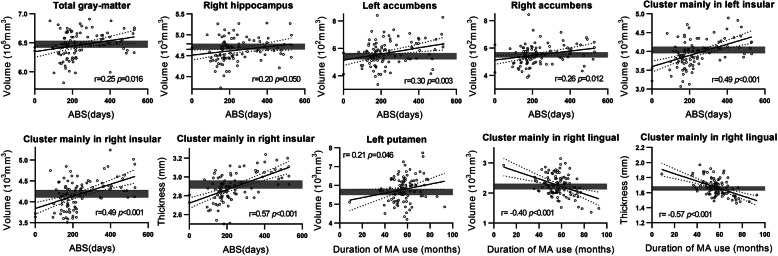
Significant correlations of subcortical and cortical measurements with duration of abstinence (ABS) and duration of methamphetamine use. Subcortical measurements were provided by Freesurfer directly while the cortical measurements were extracted from clusters shown in Table 2. Statistical methods were clarified in the notes of Table 2. The scatters were adjusted by the covariates same as that were controlled in partial correlations. Gray bars showed 95% CI of the healthy controls

Duration of abstinence was positively correlated with the volume of total gray-matter, right hippocampus and bilateral accumbens nuclei, and of clusters in bilateral insulae extending to inferior parietal lobe (Table 2, Figs. 1, 2, and Supplementary Table 1). No negative correlation was evidenced.

Duration of abstinence was positively correlated with the thickness of a cluster in right insula extending to superior temporal gyrus (Table 2, Figs. 1 and 2).

### Effects of duration of methamphetamine use

Duration of methamphetamine use was positively correlated with the volume of left putamen and negatively correlated with a region in right lingual extending to pericalcarine (Table 2, Figs. 1, 2, and Supplementary Table 1).

Duration of methamphetamine use was negatively correlated with the thickness of a cluster in right lingual extending to pericalcarine (Table 2, Figs. 1 and 2).

### Effects of onset age of methamphetamine use

Onset age of methamphetamine use showed no correlation with volumes of subcortical gray-matter structures, and with the volumes and thicknesses of the cortex.

## Discussion

This study identified structural (volume and thickness) alternations of gray-matter in chronic methamphetamine users who have accepted supervised abstinence for 8 months on average. The effects of duration of methamphetamine use, duration of abstinence and onset age of methamphetamine use within methamphetamine users were also investigated. Only a very small proportion of users have ever used limited types and very small proportion of addictive substances other than methamphetamine (Table 1). Especially, they never used marijuana and opiates. Smoking was at a very low level and the alcohol was completely forbidden during the abstinent period. Therefore, this study has an advantage to delineate the effects of single methamphetamine rather than mixed effects of multi substances. And, the abstinence was supervised and lasted to a long period which qualified the observation of the effects of long-lasting abstinence. Additionally, our large sample size allows to find more reasonable effects.

In this study, group comparisons identified greater thickness in users in bilateral superior frontal gyri, which is casually consistent with findings of gray-matter increment in frontal areas in a mixed sample of youngers with creational use of methamphetamine and cocaine [7]. Consistent with a previous study [8], this study also identified smaller volume in right lateral occipital region in users. Within these significant clusters, no reliable correlation of thickness and volume with duration of abstinence, duration of methamphetamine use and onset age of methamphetamine use was identified. Therefore, these findings were considered as reflecting either a pre-existing deficit in methamphetamine users or a lack of adaptation to an insult from methamphetamine. Although the design of the present study gave low power for identifying pre-existing conditions, we still preferred the latter. That is because a previous study has identified greater amygdala, putamen and smaller postcentral gyrus, insula, and superior temporal gyrus as pre-existing conditions of stimulants users by observing the user’s healthy siblings [19]. Those regions did not overlap with the present significant regions.

The present study also observed that the duration of abstinence was positively correlated with the volume (and with thickness for right insula) of total gray-matter, right hippocampus, bilateral accumbens nuclei and of clusters mainly in bilateral insulae extending to left inferior parietal lobe and right superior temporal gyrus. Providing that correlation with duration of abstinence reflects the effects of releasing from the pressure of methamphetamine, these positive correlations implicate that abstinent users were experiencing a recovery from methamphetamine induced gray-matter reduction in volume and thickness. The effects of methamphetamine use suggested by these correlations generally agree with previous findings of volumetric reductions in hippocampus [9], right inferior parietal lobe and left superior temporal gyrus [11, 15, 20], but disagree with previous findings of gray-matter increment in putamen [7, 8, 13, 14], nucleus accumbens [8, 13], caudate [13] and globus pallidusin [13, 14], and disagree with previous findings of gray-matter loss in limbic cortex [9], cerebellum [7] and frontal lobe [11, 12, 16]. It is not probably the variation in duration of abstinence induces these discrepancies, because with such a large sample size, we didn’t identify reliable correlation of duration of abstinence with volume of these structures or regions. On the other hand, multi-drug use might be one of the most important reasons inducing these inconsistencies. Other widely abused addictive substances, such as opiates and marijuana, could induce gray-matter reduction in pre- and orbitofrontal gyri, insulae and temporal cortex [21, 22]. These regions are highly overlapped with previous findings in methamphetamine dependent samples for which the effects of other substances were not excluded explicitly [11, 12, 16].

Structural alternations in the brain would have functional implications. The superior frontal gyrus participates in the regulation of emotions [23, 24] and working memory [25] which include the short-term maintenance of relevant information, the mental manipulation of this information and the mental organization of the forthcoming sequence of actions [26, 27]. The insula cortex plays an integral role in executive functions, as well as processing motivational states [28, 29]. Specifically, it is involved in error detection [30], cognitive mediation [31], and the integration of environmental, internal, and social information to regulate behaviors [32]. Connecting by fronto-insula tracts [33], fronto-insula cortex also plays a critical role in mediating interactions between central-executive and default-mode networks [34]. Disruption to this circuitry could impair the ability to properly weigh risk versus reward for behavior and could explain their propensity to have more errors and poorer task monitoring on neuropsychological testing and disrupted cognitive control [35] and thus impairs decision-making that is guided by subjective responses to somatic and external cues [36]. The findings of persistent greater thickness in bilateral superior frontal gyri further support these functional implications. Methamphetamine-dependent subjects do not show considerable cognitive gains in the first month of abstinence [37], and not all the impairment is recoverable along with prolonged abstinence (13-month on average), such as verbal, learning and memory, executive functions [38] which are related to the function of superior frontal gyrus. In recent years, accumulating evidences have shown that repetitive transcranial magnetic stimulation (rTMS) over dorsal-lateral prefrontal cortex is beneficial for methamphetamine dependents by alleviating craving, withdrawal symptoms, depression and anxiety [39–42], and by improving sleep quality [39], verbal learning and memory and social cognition [42]. These results may help to support studies of whether deep brain or transcranial stimulation over the altered regions is beneficial for methamphetamine-induced neurocognitive disfunctions.

It is difficult to delineate the underling mechanisms for these alternations. Methamphetamine produces long-term damage to dopaminergic and serotonergic axon terminals in the striatum, hippocampus, and prefrontal cortex [5]. All the altered gray-matter structures identified by this study accept dopaminergic projections, and the affected regions in cortex were located in midline and lateral areas that is coincide with the distribution of dopamine receptors [43], which implicate that these particular alternations are at least partly secondary to the effects of abnormal dopamine release induced by methamphetamine. However, the altered regions accepted only part of the dopaminergic projections from either the ventral tegmental area or the substantia nigra pars compacta, which implicates that these particular alternations depend less on where the dopaminergic projection comes [5], but more on, for example, the density of dopamine receptors, of this particular or relevant locations. It has been proposed that dopamine D1 receptors are major modulators of synaptic plasticity in the frontal cortex [44], additionally, previous studies have evidenced that midbrain D2/D3 [45] and striatal D1 [46] receptors modulate gray-matter adaption in chronic methamphetamine users.

Considering that various mechanisms underlie this regional specific responding of gray-matter to chronic methamphetamine use is reasonable. The permanent greater thickness in bilateral superior frontal gyri is most likely due to gray-matter gliosis induced by chronic methamphetamine use [3]. As to regions showing recoverable alternations in volume or thickness of regions around bilateral insulae, it might simply reflect the recovery from abnormal dopamine release. The hippocampus is one of the only two regions which have the neuron regeneration [47, 48], indicating that this mechanism would contribute to the recovery effect identified in this region. Histological study is needed to further clarify the regional specific response of gray-matter to methamphetamine. It should be noted that this study didn’t identify gray-matter alternation in ventral tegmental area and substantia nigra pars compacta where the dopaminergic neurons are located. It is likely that the origins of dopaminergic and serotonergic neurons are too small for MRI to detect.

This study additionally identified a negative correlation of volume/thickness in right occipital cortex and a positive correlation of volume of left putamen with duration of methamphetamine use, which suggests that the chronicity of methamphetamine use influenced the effects of methamphetamine on gray-matter. However, it is very interesting that the significant region showed no group difference and showed no correlation with abstinence. The design, sample size and not reliable detailed methamphetamine use history of this study limited us to further investigate the effects of variations in pattern of methamphetamine use on gray-matter structures. Additionally, although previous studies suggest differences between adolescence and adulthood in reaction of the central dopaminergic system to methamphetamine [49], onset age of methamphetamine use showed no effects in the present study. Therefore, effects of methamphetamine on developing brain needs to be studied extensively.

This study has several limitations. First, this study is not a longitudinal one. It could not directly discriminate the effects of cumulative effect of methamphetamine and abstinence on gray-matter, but to illustrate their correlations. To some extent, it reduces the sensitivity. Second, poly-drug use could not be completely scrolled out. Third, although those users were abstinent from cigarette and alcohol at present, we could not completely exclude history effects because of the absence of detailed information about history use. However, the users are young, so it is expected that there’s less influence. It should be noted here that it is likely our participants used more amount of methamphetamine than recreational users as they were under compulsory abstinent in Compulsory Drug Addiction Treatment Agencies and used less than participants in most of the previous studies as other studies always use dependence as the inclusion criteria. This might be an additional factor leading to the inconsistency between our study and others’.

## Conclusions

Chronic methamphetamine use induces hard-to-recover cortical thickening in bilateral superior frontal gyri and recoverable volumetric reduction in right hippocampus, bilateral accumbens nuclei and bilateral cortical regions around insulae. These alternations might contribute to methamphetamine-induced neurocognitive disfunctions and reflect a regional specific response of the brain to methamphetamine. Additionally, chronicity of methamphetamine use showed effects, suggesting that variation in pattern of methamphetamine use would influence the effects of methamphetamine. However, this part of result is not sufficient for a decisive conclusion. Longitudinal studies with samples of pure methamphetamine users are guaranteed.

## Supplementary information

**Additional file 1.**

## Data Availability

The datasets used during the current study are available from the corresponding author on reasonable request.

## Abbreviations

MA: Methamphetamine
MDMA: Methylenedioxymethamphetamine
ABS: Duration of abstinence from methamphetamine
SCID: Structured clinical interview from the diagnostic and statistical manual of mental disorders, fourth edition
MRI: Magnet resonance imaging
MPRAGE: Magnetically prepared rapid acquisition gradient echo
TR: Repetition time
TE: Echo time
TI: Inversion time
FOV: Field of view
ANCOVA: Analysis of covariance
CSF: Cerebrospinal fluid
ICV: Total intracranial volume
