# Author Correction to: Gray-matter structure in long-term abstinent methamphetamine users

**DOI:** 10.1186/s12888-021-03250-x

**Published:** 2021-06-09

**Authors:** Lili Nie, Zeyong Zhao, Xiantao Wen, Wei Luo, Tao Ju, Anlian Ren, Binbin Wu, Jing Li

**Affiliations:** 1 Mental Health Center, West China Hospital of Sichuan University, Chengdu, 610041 China; 2 Detoxification and Narcotics Control Department of Sichuan Province, Chengdu, 610041 China; 3 Sichuan provincial Compulsory Drug Addiction Treatment Agency for Males, Ziyang, 641400 China; 4 Sichuan provincial Compulsory Drug Addiction Treatment Agency for Females, Deyang, 618007 China; 5 Hospital of Sichuan provincial Compulsory Drug Addiction Treatment Agency for Females, Deyang, 618007 China

**Correction to: BMC Psychiatry 20, 158 (2020)**

**https://doi.org/10.1186/s12888-020-02567-3**

Following publication of the original article [1], the authors would like to make some clarifications.

**Clarification**

In the publication “*Gray-matter Structure in Long-term Abstinent Methamphetamine Users*”, we identified the effects of chronic methamphetamine (MA) use on gray-matter structure with a sample of which little proportion had ever used limited types and limited amounts of drugs other than MA. Due to concerns regarding gender differences in clinical characteristics of the users, and whether the present findings were distorted by polydrug users, we now provide additional information of clinical characteristics of the users, and additional analysis to prove that polydrug users did not distort the present findings.

**Methods**

We now provide descriptive information for gender differences in clinical characteristics of MA users, and two lines of analysis on gray-matter structures additionally.

First, we performed whole-brain scale vertex-wise comparisons on cortical thicknesses of 1) all users (*n* = 99) vs. healthy controls(*n* = 86), 2) nominally pure users(*n* = 72) vs. healthy controls, and 3) mixed samples (randomly replaced 27 pure users with polydrug users, n = 72) vs. healthy controls. In accordance with the statistical method used in the publication version, all the additional comparisons were controlled for age, gender and total intracranial volume. The preprocessing of the MRI data was conducted in Freesurfer (5.3), while these additional statistical analyses were conducted in Freesurfer (6.0).

Second, we extracted the mean values of each of the significant clusters (all users vs. healthy controls), and then directly compared it in polydrug users(*n* = 27) with that in nominally pure users. Complying with the statistical method used in the publication, we built liner models with all the participants (99 users and 86 healthy controls) to regress out the effects of covariates. After that, we compared the residuals of the two groups (polydrug users vs. pure users) with *t* test.

**Results**

**Gender differences in MA users** (Table 1)

**Table 1:** Additional detailed gender difference in clinical characteristics of methamphetamine users

	Male *N* = 51	Female *N* = 48	*t*	*p*
Age at time of study (years old)	28.69 ± 7.56	25.10 ± 3.65	3.034	0.003
Duration of MA use (months)	50.75 ± 39.46	63.49 ± 30.08	− 1.814	0.073
Duration of abstinence (days)	109.98 ± 114.21	380.13 ± 131.58	−10.927	<0.001
Age at onset of MA use (years old)	24.29 ± 8.07	19.042 ± 2.90	4.361	<0.001

Gender differences were seen in *Age at time of study*, *Age at onset of MA use*, and *Duration of abstinence* (in Table 1). In other words, for users in Compulsory Drug Addiction Treatment Agencies (CDATAs), females were younger, initiated MA use earlier, and had experienced a longer period of abstinence than males.

**Additional analysis showing that the findings were not distorted by polydrug users**

The first line of results: the original statistical (*p*) maps of the following comparisons on cortical thicknesses were similar to each other (See Fig. 1). 1) all users vs. healthy controls, 2) nominally pure users vs. healthy controls, and 3) mixed samples vs. healthy controls. Here, all the clusters (vertex-wise *p* < 0.05) in maps of nominally pure users vs. healthy controls, and mixed samples vs. healthy controls did not survive under correction for multiple comparisons.

**Fig. 1:**
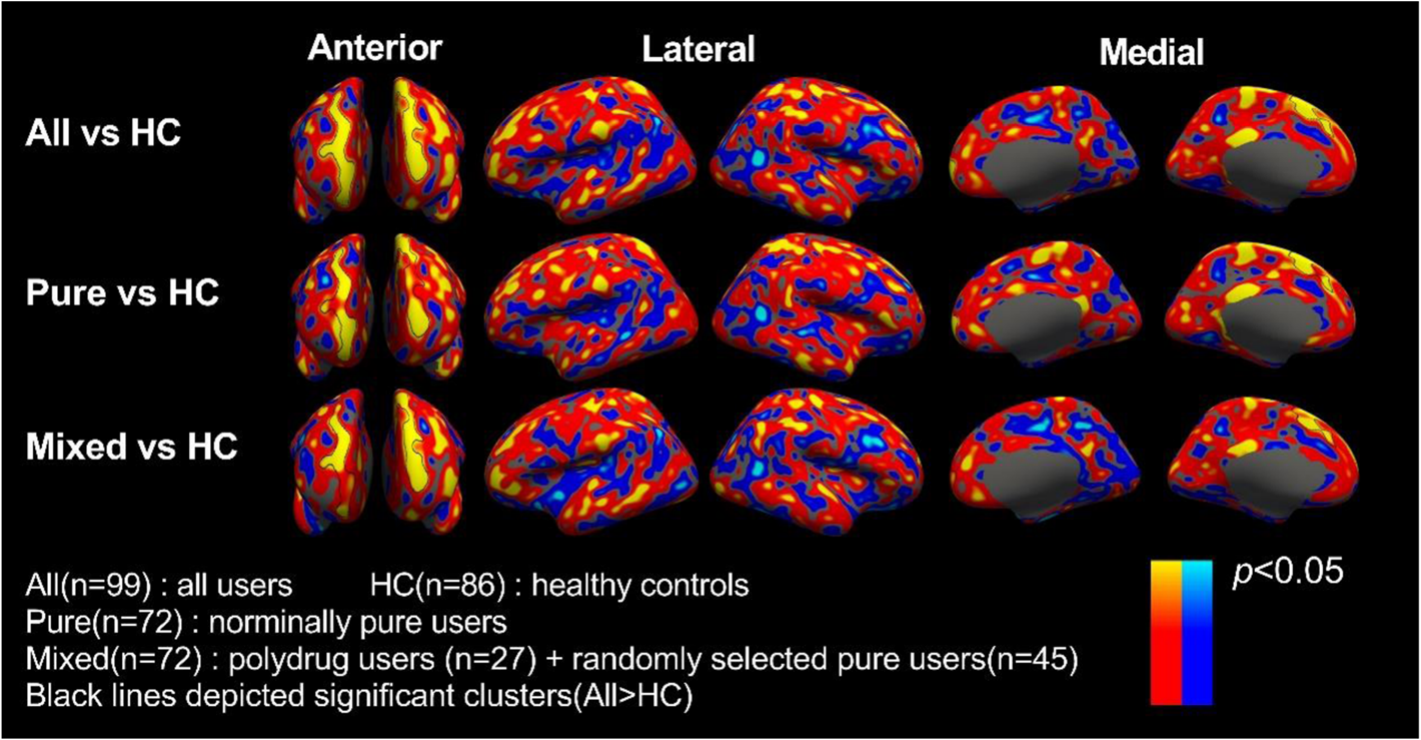
Original statical (*p*) maps of three comparisons on cortical thicknesses. Black lines in all the three rows of maps depicted significant clusters in the comparison of All vs. HC. Differences in Pure vs. HC, and Mixed vs. HC could not survive under multi-comparisons correction

The second line of results: polydrug users did not differ with pure users in the mean values of each of the extracted clusters (Table 2. pure users *n* = 72 vs. polydrug users *n* = 27, two clusters, *p* = 0.32, and *p* = 0.96 respectively).

**Table 2:** Mean thickness (mm) of clusters showing significant vertex-wise between-group difference

	HC *N* = 86	Polydrug users *N* = 27	Pure users *N* = 72	Polydrug vs. Pure	
				*t*	*p*
LH_sig_cluster	3.003 ± 0.197	3.08 ± 0.18	3.04 ± 0.16	−1.00	0.32
RH_sig_cluster	2.586 ± 0.159	2.62 ± 0.15	2.62 ± 0.18	0.46	0.96

**Discussion**

In Table 1, gender differences were shown in clinical characteristics of MA users, which possibly reflected the nature of MA prevalence in China. Firstly, our recruitment of MA users followed the principle of randomness. Secondly, we referred to another database which included 525 MA users (345males and 180 females) that belonged to another study also conducted by us aiming to identify risk factors for MA related psychosis [2]. Similar gender differences in clinical characteristics between the two study cohorts suggest that the gender differences in the present MA users reflected the real world.

All the users in the 525 cohort had ever used MA and most of them (90.86%) were polydrug users. The averaging *Age at time of stud*y of the Males (*n* = 345, 31.7 years old) in the 525 users was 4 years elder than that of the Females (*n* = 180, 27.7 years old). In comparison, male MRI users in this study were elder than females for nearly 3 years (See Table 1). *The Age at onset of MA use* (not *the onset of drug use*) of the Males (mean = 25.3 years old) in the 525 users was 4 years elder than that of the Females (mean = 21.3 years old). In comparison, *Age at onset of MA use* for male MRI users was elder than females for 5 years (See Table 1). The averaging *Duration of abstinence* of the Males (279 days) in the 525 users was shorter than that of the Females (424 days) for 210 days. In comparison, that of the MRI male users (110 days) was shorter than MRI female users (380 days) for 270 days. Very similar intervals of the three variables between the two cohorts suggest that gender differences in the MRI cohort reflect the natural world of the MA users in CDATAs. Due to these gender differences, necessary variables were controlled during the analysis on gray-matter structures. Further, extensive studies are needed to investigate the gender differences in effects of MA use and abstinence on brain structures.

Moreover, our results of additional analysis verified that polydrug users did not distort the original findings.

Whole-brain scale vertex-wise analysis (as shown in Fig. 1) showed that the statistical maps of pure users and comparable number of mixed users (pure users combined with polydrug users) respectively versus healthy controls were similar to that of all users. Additionally, values of significant clusters of pure users, polydrug users, and healthy controls did not statistically differ with each other (Table 2). The sample size of polydrug users (*n* = 27) was relatively large. Therefore, although the *t* tests were not non-inferiority design, it still didn’t support the view that the published results were distorted by the polydrug users.

Additionally, clusters that showed vertex-wise *p*<0.05 in pure users and mixed users versus healthy controls did not survive under correction for multiple comparisons, and mean values of significant clusters in all users just slightly differed with that in healthy controls, which suggested that the effect of MA on brain structures were milder. Therefore, a relatively large sample size(n=99) was needed to identify it, just as in the present sample.It should be noted that, we have conducted a ROI based analysis with a subset of nominally pure users (*n* = 61) to identify whether measurements of gray-matter structures of several regions of interest (ROIs) correlated with the duration of abstinence [3]. The chosen of ROIs were based on previous studies which identified gray-matter structural alternations in MA dependent participants. It was mainly found that abstinence from MA resulted in volumetric increases in regions that were important for cognitive function including orbitofrontal and parietal cortices and hippocampi, while male users had smaller hippocampi than male controls. However, the present study did not evidence vertex-wise difference in the ROIs suggested by previous studies when compared with healthy controls. Extensive studies are guaranteed to identify interactions between MA and other substances on their effects on gray-matter structures, especially in these tested ROIs.

In conclusion, gender differences in clinical characteristics of MA users reflect the reality of MA use in China while necessary variables had been properly controlled in the data analysis. Besides, present evidences did not support that the published results were obviously distorted by polydrug users.

## References

[1] Nie L, Zhao Z, Wen X, Luo W, Ju T, Ren A, Wu B, Li J (2020). Gray-matter structure in long-term abstinent methamphetamine users. BMC Psychiatry.

[2] Nie L, Zhaom Z, Wen X, Luo W, Ju T, Ren A, Wu B, London ED, Li J (2018). Factors affecting the occurrence of psychotic symptoms in chronic methamphetamine users. J Addict Dis.

[3] Nie L, Ghahremani DG, Mandelkern MA, Dean AC, Luo W, Ren A, et al. The relationship between duration of abstinence and gray-matter brain structure in chronic methamphetamine users. Am J Drug Alcohol Abuse. 2021:1–9. 10.1080/00952990.2020.1778712 33426968

