# Supplementary material for: Gray-matter structure in long-term abstinent methamphetamine users
Source: BMC Psychiatry. 2020 Apr 10;20:158. doi: 10.1186/s12888-020-02567-3 (PMC7146984; doi:10.1186/s12888-020-02567-3)
Supplement: Supplementary file 1 — Additional file 1. [file 12888_2020_2567_MOESM1_ESM.docx]

**Statistical methods**

When we tested the effects of methamphetamine use (group comparisons) and subsequently abstinence (correlation with duration of abstinence) on structural measurements (thickness and volume) of the gray-matter, total intracranial volume (ICV) was controlled whereas education was not.

ICV influences the size of the brain which contributes to the morphological characteristics of certain gray-matter structures [1]. In this study, methamphetamine users showed smaller ICV than healthy controls (HCs) (supplementary results and table 1), which, as we have mentioned in the supplementary discussion, suggests that the users exhibited pre-existing developmental variants that should be controlled in identifying the effects of methamphetamine. Thickness and volume are commonly used in studies on the cortex of the brain, and the volume of cortical gray-matter reflects both thickness and area [2]. Therefore, ICV was controlled in all the analyses on thickness and volumes.

Although years of education showed significant group difference in this study, it was not controlled in all the analyses because controlling for education would induce noises.

In line with a previous report [3], finding of positive correlation between duration of education and ICV in healthy controls (supplementary results) seems to suggest that education reflects a developmental variation which could contribute to morphological characteristics of gray-matter structures. Therefore, it should be controlled in the analyses. However, within our knowledge, no study supports the view that general education influences morphological characteristics of gray-matter structures. Consistent with this view, we didn’t identify correlation of education with total gray-matter volumes (supplementary results). Additionally, correlation of education with ICV was absent in users, which suggests that education in users did not reflect similar developmental variant as in healthy subjects. Schwartz et.al. have argued that less education (than healthy subjects) in users reflects a drop-out which is affected by drug use [4]. Therefore, using education as a covariate would induce noises by adjusting the data depending on a linear relationship formed mainly by the healthy controls.

**Results**

ICV of methamphetamine users (1.48±0.13, 10^6^mm^3^) was significantly smaller than that of healthy controls (1.54±0.13, 10^6^mm^3^). Within healthy controls, the years of education was positively correlated with ICV (r=0.231, *p*=0.034, controlling for sex and age), but not with total gray-matter volume (r=0.156, *p*=0.156). Within the users, neither ICV (r=0.050, *p*=0.629) nor total gray-matter volume (r=0.009, *p*=0.931) showed correlation with years of education when controlling for sex and age.

**Discussion**

In this study, methamphetamine users showed smaller ICV than healthy controls (supplementary table 1). The ICV of human beings reaches the maximum at 16-17 years old [5]. The first use of methamphetamine users in this study was at a mean age of 21.73 ± 6.65 years old, suggesting that most of the users started to use after their ICV had reached the maximum. Therefore, smaller ICV in users reflects a developmental variation rather than the effects of methamphetamine. One previous study on siblings of stimulants users identified greater amygdala, putamen and smaller postcentral gyrus, insula, and superior temporal gyrus, which supports the view that the brain of methamphetamine users exhibits pre-existing morphological characteristics [6]. However, the design of the present study limited us to further clarify this question.

**References**:

1. Jancke L, Merillat S, Liem F, Hanggi J: **Brain Size, Sex, and the Aging Brain**. *Human brain mapping* 2015, **36**(1):150-169.

2. Winkler AM, Kochunov P, Blangero J, Almasy L, Zilles K, Fox PT, Duggirala R, Glahn DC: **Cortical thickness or grey matter volume? The importance of selecting the phenotype for imaging genetics studies**. *NeuroImage* 2010, **53**(3):1135-1146.

3. Mortimer JA, Snowdon DA, Markesbery WR: **Head circumference, education and risk of dementia: findings from the Nun Study**. *J Clin Exp Neuropsychol* 2003, **25**(5):671-679.

4. Schwartz DL, Mitchell AD, Lahna DL, Luber HS, Huckans MS, Mitchell SH, Hoffman WF: **Global and local morphometric differences in recently abstinent methamphetamine-dependent individuals**. *NeuroImage* 2010, **50**(4):1392-1401.

5. Purkait R: **Growth of cranial volume: an anthropometric study**. *J Plast Reconstr Aesthet Surg* 2011, **64**(5):e115-117.

6. Ersche KD, Jones PS, Williams GB, Turton AJ, Robbins TW, Bullmore ET: **Abnormal brain structure implicated in stimulant drug addiction**. *Science* 2012, **335**(6068):601-604.

**Supplementary Table 1 Volumes of subcortical gray-matter structures (mm^3^)**

|  | Group Comparisons **^a^** | | | | ABS **^b^** | | MA use **^c^** | |
| --- | --- | --- | --- | --- | --- | --- | --- | --- |
|  | Control | MA | F | *p* | r | *p* | r | *p* |
| **Total intracranial volume** | 1543180±131369 | 1478872±128774 | 12.75 | **<0.001** | 0.12 | 0.23 | 0.07 | 0.52 |
| **Total gray-matter** | 658356±54103 | 637227±49897 | 0.08 | 0.78 | 0.25 | **0.016** | 0.10 | 0.36 |
| Thalamus L | 8684±881 | 8290±854 | 1.04 | 0.31 | -0.05 | 0.66 | 0.01 | 0.94 |
| Thalamus R | 7643±718 | 7361±725 | 0.45 | 0.50 | -0.01 | 0.96 | -0.01 | 0.93 |
| Caudate L | 3677±458 | 3605±458 | 0.89 | 0.35 | -0.07 | 0.48 | 0.05 | 0.63 |
| Caudate R | 3516±482 | 3486±492 | 1.98 | 0.16 | 0.01 | 0.96 | 0.02 | 0.87 |
| **Putamen L** | 5758±659 | 5744±762 | 1.46 | 0.23 | 0.05 | 0.65 | 0.21 | **0.046** |
| Putamen R | 5529±557 | 5422±648 | 0.21 | 0.65 | 0.15 | 0.15 | 0.15 | 0.16 |
| Pallidum L | 1344±228 | 1329±237 | 0.05 | 0.82 | 0.05 | 0.61 | 0.01 | 0.90 |
| Pallidum R | 1527±197 | 1484±237 | 0.01 | 0.95 | 0.00 | 0.99 | 0.09 | 0.41 |
| Hippocampus L | 4586±393 | 4438±345 | 0.44 | 0.51 | 0.14 | 0.18 | -0.08 | 0.44 |
| **Hippocampus R** | 4785±427 | 4577±358 | 2.79 | 0.10 | 0.20 | **0.050** | 0.06 | 0.59 |
| Amygdala L | 1777±203 | 1693±184 | 1.57 | 0.21 | 0.10 | 0.36 | -0.01 | 0.91 |
| Amygdala R | 1828±214 | 1770±204 | 0.01 | 0.91 | 0.09 | 0.39 | -0.06 | 0.59 |
| **Accumbens L** | 549±99 | 564±96 | 3.07 | 0.08 | 0.30 | **0.003** | 0.03 | 0.74 |
| **Accumbens R** | 559±85 | 546±81 | 0.02 | 0.88 | 0.26 | **0.012** | 0.06 | 0.55 |
| Ventral diencephalon L | 3970±392 | 3818±376 | 0.64 | 0.43 | -0.02 | 0.81 | -0.13 | 0.20 |
| Ventral diencephalon R | 3860±332 | 3744±376 | 0.08 | 0.78 | 0.03 | 0.76 | -0.09 | 0.41 |
| Cerebellum cortex L | 54679±5174 | 53116±5143 | 0.48 | 0.49 | -0.09 | 0.39 | 0.10 | 0.34 |
| Cerebellum cortex R | 55833±5758 | 53798±5454 | 1.19 | 0.28 | -0.14 | 0.18 | 0.12 | 0.25 |

**^a^** Data were shown as mean ± SD. Group comparisons were using analysis of covariance (ANCOVA) with sex, age and total intracranial volume (ICV) controlled, except for the comparison of ICV, which was only controlled for sex and age.

**^b^** Partial correlations with sex, age, age at onset of methamphetamine use, duration of methamphetamine use and ICV controlled.

**^c^** Partial correlations with sex, age, age at onset of methamphetamine use, duration of abstinence and ICV controlled.

ABS: duration of abstinence from methamphetamine. MA: methamphetamine. MA use: duration of methamphetamine use.

The *p* values were not corrected for multiple comparisons.
